# Are Religious Practices Indirectly Related to Stress at Work Through the Tendency to Forgive? A Sample of Polish Employees

**DOI:** 10.1007/s10943-022-01710-6

**Published:** 2022-12-01

**Authors:** Marcin Wnuk

**Affiliations:** Department of Work and Organizational Psychology, Adam Mickiewicz University in Poznań, Ul. Szamarzewskiego 89/AB, 60-568 Poznan, Poland

**Keywords:** Polish employees, Religious practices, Prayer, Mass attendance, Forgiveness, Stress at work

## Abstract

Religiosity has been a neglected factor in studies regarding the workplace in comparison to spirituality. Some available studies have indicated positive outcomes of religious commitment and intrinsically religious-oriented employees. There is however a lack of research explaining how religious commitment is related to occupational well-being. This study aimed to examine the mechanism of the relationship between religious practices and stress at work and the role of forgiveness as a moral virtue underlying this link. The participants in the study were 754 employees from Poland. The research used a cross-sectional design. The mechanism controlled for gender, denomination, age, education, and the level of position held, and the indirect relationship between prayer and mass attendance and stress at work through forgiveness was confirmed. Religious practices were positively related to a lack of revenge and avoidance motivation, which, in turn, was negatively correlated with stress at work. The role of religious commitment in occupational well-being was discussed, considering socio-cultural conditioning, and the theoretical and practical implications were presented.

## Introduction

Links between religiosity and mental health and well-being, despite having grown in researchers’ interest, are still an unrecognized area of research that requires further exploration. Results regarding the relationships between both phenomena have been complex depending on moderators, such as religious measures used (Garssen et al., 2021; Hackney & Sander’s, 2003; Wnuk, 2021a), socio-cultural context (background) (Lavrič & Flere, 2008; Lun & Bond, 2013; Stavrova et al., 2013), or religious denomination (Ghazzaw et al., 2016).

Also, many mediators are tested in looking for mechanisms responsible for the influence of religiosity on well-being. Researchers have tried to explain how religiosity is related to well-being, searching for strengths of character and moral virtues, such as gratitude, hope, optimism, and compassion, as factors underlying these links (Park, 2007). According to Stewart et al. (2016), literature review, gratitude, hope, and forgiveness, as a result of religious sources, may improve general well-being, health, and pro-social and positive behavior.

 Further, in the spirit of positive psychology, Watts et al. (2006) emphasized the positive role of religion from the perspective of psychological practice in facilitating hope, gratitude, and forgiveness in the way of achieving fulfillment, integrity, and a positive state of mind. Recent studies have confirmed the influence of religiosity on well-being and the significant role played by hope (Chang et al., 2013, 2016; Marques et al., 2013; Wnuk & Marcinkowski, 2014; Wnuk, 2021b), gratitude (Jang et al., 2018; Kane et al., 2021; Szcześniak et al., 2019; Van Cappellen et al., 2016), and forgiveness (Jang, et al., 2018; Kane et al., 2021; Lawler-Row, 2010; Olawa et al., 2022). For example, in a sample of Chilean students, prayer and mass attendance were indirectly related through spiritual experiences and hope to subjective well-being (Wnuk, 2021a, 2021b). In a Kane et al. study (2021) conducted among Latter-Day Saint Polynesian Americans, religious commitment, both directly and indirectly via gratitude, was positively related to self-esteem and linked only directly through forgiveness with this variable.

Religiosity is a topic neglected in human resource management (Gebert et al., 2014) and demands exploration to verify the beneficial or detrimental (harmful) effects of religion on employees’ well-being (Obregon et al., 2021; Heliot et al., 2019). Religion, especially in intrinsically religious-oriented individuals, for whom religion is a crucial aspect of their worldview and the primary value in the axiological system (Allport & Ross, 1967), positively impacts one’s attitude toward work (Kutcher et al., 2010). Some suggestions regarding this issue have been contained in Wnuk’s (2022) research, which has confirmed that prayer and being strongly connected with God are positively related to organizational gratefulness and finding meaning at work. Also, in a sample of Australian Muslim men, religious commitment influences work-life conflict, job demands, and work flexibility (Sav, 2019). In another study, intrinsic religiosity protected workers from the negative influence of burnout on job satisfaction (Bal & Kökalan, 2021).

Current research attempts to fill the gap in religiosity-occupational well-being associations by examining the relationship between religious practices and stress at work, and the role of a tendency to forgive as a variable underlying this connection.

## Literature Review

Despite the lack of research about the role of religion in the workplace, some studies have confirmed a beneficial role for positive behaviors at work (Obregon et al. 2021; Qureshi & Shahjehan, 2021), organizational commitment (Walker, 2013), work engagement (Bickerton & Miner, 2021; Zahrah et al., 2017), job performance (Zahrah et al., 2017), and coping with stress (Cunningham, 2014; Kutcher et al., 2010; Noor, 2008).

Religious commitment can influence positive outcomes at work through the disposition to forgive (Jang et al., 2018; Kane et al., 2021; Lawler-Row, 2010; Olawa et al., 2022). Despite the absence of research on the function of forgiving for an employee’s well-being, Cox et al. (2012) explored this topic by verifying some motives leading to forgiving as a potential antecedent of general health and stress. According to the results of this study, forgiving due to a moral motive was negatively correlated with stress in comparison to religious conditioning for forgiving and a lack of alternatives, which was related to a higher level of stress.

It is important to note that forgiveness as a moral virtue can have its roots in religion. On the one hand, for religiously inclined people, moral virtues, due to religion, can be treated as instrumental values. On the other hand, believers with intrinsic religious orientation perceive forgiveness as an autotelic value unconnected with coercion to act coherently with this virtue. For example, in Wnuk’s research (2021), the strength of religious faith was negatively related to motivation to avoid transgressors in a group of students who participated in Mass and used positive religious coping with an average or greater-than-average frequency. Another interpretation of Cox et al.’s (2012) results is based on religion as a way of coping through forgiveness among individuals with the highest level of stress.

Also, in Toussaint et al.’s (2018) study, the role of forgiveness in workplace outcomes was explored, showing that forgiveness as a trait is inversely correlated with unproductivity and physical and health problems, and workplace interpersonal stress partially mediated these relationships.

Forgiveness is considered an emotion-focused coping style to reduce a stressful reaction to a transgression (Worthington & Scherer, 2004) thanks to the appearance of positive emotions during the process of forgiveness (Fredrickson, 2001).

In Christianity, being forgiven is learned through imitation of the characters from the Bible or following the role models in family and work within the social learning process (Bandura, 1977), especially in backgrounds permissive for religious socialization (Lun & Bond, 2013), like Poland. This has been confirmed by the results of the research. For example, in Hardy et al., (2014) longitudinal study, daily religiosity through daily spiritual experiences was indirectly related to growing forgiveness, gratitude, and empathy (Hardy et al., 2014). Also, in a sample of undergraduate students, intervention based on prayer led to an increase in the state of forgiveness compared to the control group (Vasiliauskas & McMinn, 2013).

Religious commitment can be a factor that serves interpersonal forgiveness in the workplace as an effective stress-coping style. Among individuals with a strong religious-oriented system (ROS) (Pargament, 1997), being a part of a more global-orienting system, internalized religious values and rules impact daily life at work, determining their perception, evaluation, behaviors, and how they cope with stress from the religious meaning perspective. According to PEW statistics, in Poland, 66% of individuals are religiously active, which is second in the world after South Africa; among all European countries, Poland is seventh in reference to the importance of religiosity in life. Poles in Europe most frequently attend religious services.

Considering Poland’s socio-cultural context (Lavrič & Flere, 2008; Lun & Bond, 2013; Stavrova et al., 2013), openness to religious socialization and the high national rate of religiosity should determine a positive relationship between employee religiosity and well-being.

In Poland, some studies have confirmed the benefits of religiously motivated forgiveness for well-being in a sample of students (Głaz, 2019), adults (Zarzycka & Krok, 2021) and participants of Sexaholics Anonymous diagnosed with compulsive sexual behavior disorder (Wnuk & Charzyńska, 2022). For example, in Wnuk and Charzyńska (2022) study, subjective religiosity was positively related to feeling forgiven by God, which in turn through interpersonal forgiveness, was indirectly positively linked with the presence of meaning in life.

Based on these premises, it was expected that religious practices would be indirectly negatively related to stress at work through interpersonal forgiveness.

Besides self-forgiveness and God’s forgiveness, interpersonal forgiveness (Lawler-Row, 2010) is one of the forms of this phenomenon, considering the perspective of mutual relationships between people.

In this study, this construct was treated as a moral virtue (Van Tongeren et al., 2012) and moral emotion (Hardy et al., 2014), which manifested in the lack of a tendency to revenge and avoid transgressor.

## Materials and Methods

### Participants

Participation in research had an anonymous and voluntary character. The sample consisted of 754 full-time employees with employment contracts in Poland. They were not asked about the industry type they represent. All participants declared Roman Catholic denomination. Details about sociodemographic characteristics are presented in Table 1.

**Table 1:** Sociodemographic characteristics of the study sample (*n* = 754). *Source* author’s research)

Variables	N	%
*Gender*		
Men	304	40.4
Women	450	59.6
Age (years; M ± SD)	29.54 (± 10.22)	
*Educational level*		
Elementary	4	0.5
Vocational	29	3.8
Secondary	312	41.4
Higher	409	54.2
*Level the position held*		
Ordinary workers	260	34.5
Independent specialist	319	42.3
Low managers	57	7.6
Middle managers	86	11.4
Senior managers	32	4.2

### Measures

The Transgression-Related Interpersonal Motivations Scale (TRIM) is used to measure motivation to forgive (McCullough et al., 1998). This measure consists of 12 items; five of them refer to revenge, and seven refer to avoiding the transgressor. Responses are given on a 5-point Likert scale from 1 (*strongly disagree*) to 5 (*strongly agree*). This scale has acceptable reliability, measured by relative stability (revenge: Cronbach’s *α* = 0.90; avoidance: *α* = 0.86–0.94) and absolute stability measured by means of test–retest reliability with an interval of three to nine weeks (revenge: *α* = 0.53–0.79; avoidance: *α* = 0.44–0.86). The scale has acceptable internal validity, measured by means of factor analysis, as well as acceptable convergent and discriminant validity, measured by correlations with other measures of forgiveness and similar constructs (McCullough et al., 1998).

The short version of the Polish adaptation of the Perceived Stress at Work Scale (Chirkowska-Smolak & Grobelny, 2016; Cohen & Janicki-Deverts, 2012) consisting of five items was applied to verify the level of stress at work. Participants responded on a 5-point Likert scale ranging from 5 = never to 1 = almost always (Cohen & Janicki-Deverts, 2012). The good psychometrical properties of this short version tool in Polish conditions were confirmed (Wnuk, 2018, 2019). This research confirmed the one-factor structure of the short version of this measure. The five-item solution explained 63.35% variance of stress at work.

Prayer was verified through the question “How often do you pray” with the following responses: never (1), sometimes (2), once a month (3), once a week (4), and every day (5). The participants’ mass attendance was measured on the basis of a 5-point scale consisting of (1) never, with the exception of baptisms, weddings, or funerals; (2) a few times a year; (3) 1–2 times monthly; (4) 2–3 times monthly; and (5) once per week or more.

### Procedure

The participants were recruited on the internet through the information in social media where the link to access the online questionnaire was contained. After going to the survey website, there was the introductory note, which informed about the study purposes, anonymous and voluntary take a part in the research, the approximate duration of the survey, and the right to withdraw from the research at any time and without consequences. After reading the instruction, each participant was asked to agree to participate in the study by marking the appropriate box. No incentives for taking a part in the study were offered.

### Statistical Analyses

For statistical analyses, IBM SPSS statistics software (Version 27.0) and IBM Amos statistics software (Version 26.0) were applied.

Before path analysis, factor analysis was used to test the discriminant validity of the research tools and confirm that they measure separate constructs and are good indicators of them. Next, path analysis in structural equation modeling was applied as a good method to verify total, direct and indirect effects. The maximum likelihood method was used due to the results of skewness values in the range of − 2 to + 2 and kurtosis values between − 7 and + 7 (see Table 2), which suggested that the distribution of research variables is like the normal distribution (Hair et al. 2010). Multicollinearity was examined with a variance inflation factor (VIF) (James et al., 2013) and common method bias by applying Harman’s single factor (Podsakoff et al., 2003). To verify the indirect effect, the Bollen-Stine bootstrapping solution with 5.000 subsamples with 95% bias-corrected confidence interval was applied (Hayes, 2013). To recognize that the observed effects are statistically significant in every case, the bootstrapped confidence interval should not contain zero (Hayes, 2013).

**Table 2:** Descriptives statistics in a study sample (*n* = 754). *Source* author’s research

Variable	Reliability	Minimum	Maximum	Mean	Standard deviation	Skewness	Kurtosis	VIF
Mass attendance	–	1	5	2.36	1.26	0.25	− 1.61	2.4
Prayer	–	1	5	2.61	1.60	0.53	− 1.37	2.44
Lack of revenge motivation	.89	5	25	18.85	4.43	− 0.62	0.1	1.28
Lack of avoidance motivation	.89	7	35	18.83	6.19	0.47	− 0.1	1.23
Stress at work	.85	5	25	13	3.91	0.30	0.14	–
Age	–	18	77	29.38	10.15	1.48	1.29	1.23

*VIF* variance inflation factor

The following goodness-of-fit indices model was used: the root mean square error of approximation (RMSEA), the standardized root mean square residual (SRMR), the comparative fit index (CFI), the goodness-of-fit index (GFI), the normed-fit index (NFI), and the Tucker–Lewis Index (TLI).

The RMSEA cut-off value should be below 0.07 (Steiger, 2007) or, in a more restricted approach, below 0.06 (Hu & Bentler, 1999). Values of 0.95 or greater indicate a good model fit for CFI (Hu & Bentler, 1999), GFI (Hooper et al., 2008), and NFI (Hu & Bentler, 1999). Considering that in one study forgiveness only partially mediated the relationship between religiosity and well-being (Jang et al., 2018), an alternative model with direct paths between religious practices and stress at work was tested.

## Results

Descriptive statistics are presented in Table 2. Table 3 is shown the results of r-Pearson correlation coefficients between research variables.

**Table 3:** Correlation coefficients between research variables (*n* = 754). *Source* author’s research

	1	2	3	4	5	6	7	8
1.Mass attendance								
2. Prayer	0.76**							
3. Lack of revenge motivation	0.31**	0.32**						
4. Lack of avoidance motivation	0.21**	0.22**	0.39**					
6. Stress at work	0.02	0.01	0.14**	− 0.15**	0.06			
7. Age	0.10*	0.15**	0.06	0.16**	0.17**	0.03		
8. Level of position hold	0.02	0.05	0.06	0.17**	0.04	− 0.04	0.41**	
9. Education	0.06	0.08	0.10**	0.05	0.03	− 0.07	0.14**	0.29**

^*^p ≤ 0.05

^**^p ≤ 0.01

**Fig. 1:**
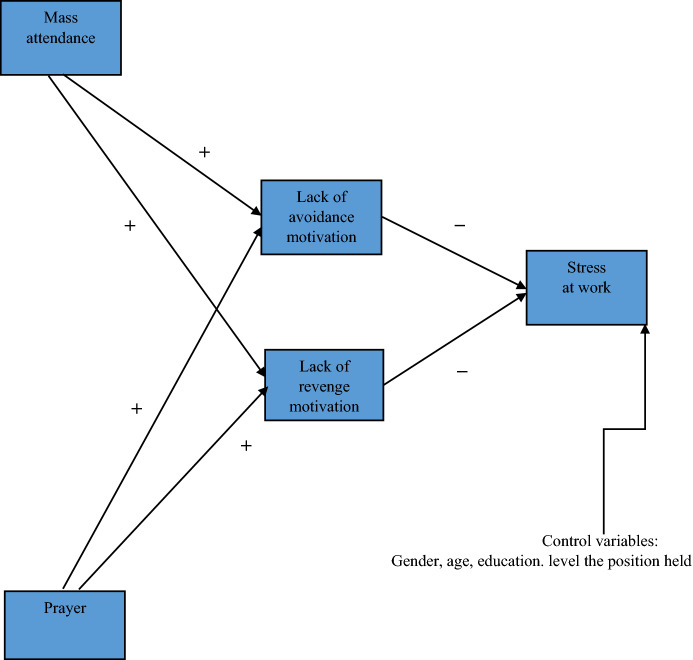
Conceptual model. *Source* author’s research

The VIF values for study variables were less than the cut-off level of 5, confirming the lack of a multicollinearity problem (James et al., 2013). Also, the result of Harman’s single factor has proven that the data were not contaminated by common method bias. The Kaiser–Meyer–Olkin test for sampling adequacy was satisfactory at − 0.870 and was higher than the minimum standard of 0.7 (Hoelzle & Meyer, 2013). Bartlett’s test of sphericity showed that the sample was properly composed (Chi2 ≈ 7697.703, df = 171, *p* < 0.01). One factor explained 28.44% of the variance, which was lower than the threshold of 40% (Podsakoff et al., 2003). Conceptual model reflecting research hypotheses is presented in Figure 1.

The results of the factor analysis confirmed the good construct validity of the measures applied in the study and satisfying discriminant validity between them. The four-factor solution explained 67.88% of the model variance. Table 4 presents the values of the items’ loading factors. All items filled the condition of the cut-off ratio for factor loading, which was 0.6, and every item assigned to one factor did not load other factors with more than a 0.2 value (see Table 4) (Henson & Roberts, 2006), excluding one item regarding avoidance motivation.

**Table 4:** Results of confirmatory factor analysis among items being a part of research measures (*n* = 754)

Items	Factors			
	1	2	3	4
Mass attendance	− .007	.008	.010	.935
Prayer	− .002	.019	− .013	.928
Stress at work	− .011	− .067	.720	− .051
Stress at work	− .003	− .006	.811	− .015
Stress at work	.027	.069	.823	.057
Stress at work	− .056	.004	.802	− .029
Stress at work	.051	− .017	.818	.030
Lack of avoidance motivation	.825	− .058	.001	− .015
Lack of avoidance motivation	.720	.071	.046	.057
Lack of avoidance motivation	.703	.121	.063	− .049
Lack of avoidance motivation	.621	.225	− .021	− .112
Lack of avoidance motivation	.873	− .071	− .045	− .013
Lack of avoidance motivation	.838	− .098	− .010	.060
Lack of avoidance motivation	.870	− .078	− .015	.027
Lack of revenge motivation	.008	.771	.022	.038
Lack of revenge motivation	.002	.848	− .003	.018
Lack of revenge motivation	.136	.767	− .036	.038
Lack of revenge motivation	.057	.893	.013	− .039
Lack of revenge motivation	− .058	.883	− .016	− .012

### Path Model Results

**Fig. 2:**
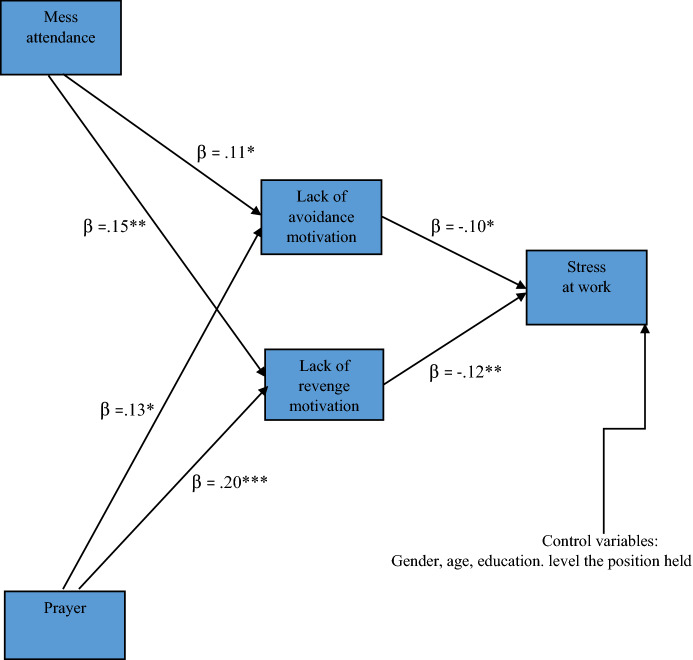
Results of path analysis. *Note* The standardized regression coefficients are presented. *p < .05, **p < .01, ***p < .001. *Source* author’s research

The direct relationships between Mass attendance and prayer with stress at work were irrelevant (95% CI [− 0.02, 0.19]; *p* = 0.119; *β* = 0.08) and (95% CI [− 0.12, 0.10]; *p* = 0.847; *β* = − 0.01), respectively. In further analysis, only the model presented in Fig. 2 with indirect effects between religious practices and stress at work was verified. The values of indices confirmed that the model was well fitted: *χ*^2^(10) = 11.92; *p* = 0.29; CMIN/df = 1.19; CFI = 0.99; GFI = 0.99; NFI = 99; TLI = 0.99; RMSEA = 0.016 (90% CI [0.000, 0.044]) SRMR = 0.023.

Results of standardized total, direct and indirect effects are presented in Table 5.

**Table 5:** Results of standardized total, direct and indirect effects for 95% interval (*n* = 754). *Source* author’s research

Pathway	Value of effect	*p*	LLCI	ULCI
*Total effect*				
Prayer—lack of avoidance motivation	0.13	0.015	0.02	0.12
Prayer—lack of revenge motivation	0.20	0.000	0.10	0.31
Mass attendance—lack of avoidance motivation	0.11	0.042	0.01	0.21
Mass attendance—lack of revenge motivation	0.15	0.005	0.05	0.25
Lack of avoidance motivation—stress at work	− 0.10	0.010	− 0.18	− 0.02
Lack of revenge motivation—stress at work	− 0.12	0.001	− .0.20	− 0.04
Prayer—stress at work	− 0.04	0.000	− .07	− .02
Mass attendance—stress at work	− 0.03	0.002	− .01	− .05
*Direct effect*				
Prayer—lack of avoidance motivation	0.13	0.015	0.02	0.12
Prayer—lack of revenge motivation	0.20	0.000	0.10	0.31
Mass attendance—lack of avoidance motivation	0.11	0.042	0.01	0.21
Mass attendance—lack of revenge motivation	0.15	0.005	0.05	0.25
Lack of avoidance motivation—stress at work	− 0.10	0.010	− 0.18	− 0.02
Lack of revenge motivation—stress at work	− 0.12	0.001	− .0.20	− 0.04
*Indirect effect*				
Prayer—stress at work	− 0.04	0.000	− .07	− .02
Mass attendance—stress at work	− 0.03	0.002	− .01	− .05

LLCI = 95% confidence interval (low); ULCI = 95% confidence interval (high)

Both mass attendance and prayer were statistically significant, directly related to the lack of revenge motivation and avoidance motivation. Also, relevant indirect effects of these variables on stress were noted. Mass attendance and prayer were indirectly related to stress through a lack of revenge and avoidance motivation.

Lack of revenge motivation and avoidance motivation was significantly directly related to stress.

Education, age, and level of position held were not statistically significantly related to stress at work (95% CI [− 0.14, 0.01]; *p* = 0.097; *β* = − 0.06), (95% CI [− 0.05, 0.11]; *p* = 0.429; *β* = 0.03), and (95% CI [− 0.07, 0.09]; *p* = 0.717; *β* = 0.01), respectively. Gender was a negative predictor of stress at work (95% CI [− 0.21, − 0.06]; *p* = 0.010; *β* = − 0.13). This was confirmed by a Student’s *t* test (*t* = 3.27; *p* < 0.001). Women (*M* = 13.37; SD = 3.93) declared more stress at work than men (*M* = 12.44; SD = 3.81).

## Discussion

This study focused on the relationship between religious practices and stress at work and the role of interpersonal forgiveness underlying this link. It was expected that, among Polish employees as a religiously committed sample, where religious socialization is prevalent, religious practices are positively directly linked with a disposition to forgive and negatively indirectly linked to stress at work. In other words, the beneficial function of prayer and mass attendance on employees’ well-being at the workplace was tested. The sample was homogeneous, considering religious denominations. Also, sociodemographic variables, such as gender, education, age, or level of position held, were controlled.

According to expectations, prayer and mass attendance positively correlated with both forgiveness indicators, such as lack of motivation for revenge and avoidance. Additional verification regarding the direct relationship between religious practices and stress at work has confirmed that these links are not statistically significant. Prayer and mass attendance were indirectly related to stress at work through forgiveness. This means that prayer as a private religious practice and mass attendance as a public worship manifestation are antecedents of a forgiveness attitude and, because of that, are indirectly favorable for stress experiences at work.

The obtained results are consistent with recent studies, which have indicated a mediating function of forgiveness in the relationship between different indicators of religiosity and a plethora of measures of well-being and mental health (Jang et al., 2018; Kane et al., 2021; Lawler-Row, 2010; Olawa et al., 2022), but with one difference regarding workplace context. As in Lawler-Row’s (2010) and Kane et al. (2021) studies, total mediation was noticed, but contrary to Jang et al. (2018) research, where partial mediation was identified, which means that besides indirect effect through forgiveness, direct effect also had a place.

Like previous research, religiosity and forgiveness were positively related (Hardy et al., 2014; Lawler-Row, 2010; Vasiliauskas & McMinn, 2013; Wnuk, 2022) independent of the used measure of both constructs. This shows that religious commitment probably has positive repercussions on forgiveness as a moral virtue in the workplace.

Religion is one of the different sources of moral norms, virtues, and values that project on the employee’s functioning in the workplace. A stronger ROS implies that they are more important in the workplace and impact behaviors because the employee wants to behave in coherency with them. This is a probable cause of a greater tendency to forgive the transgressor, and the factor facilitates perceiving the harmful situation in a more favorable way and motivates one to look for the solution to adjust. In this kind of situation, support can be teachings from the Scriptures, like Jesus’s words, where he talks about the frequency of forgiveness. Also, Jesus’s own life attitude, especially his approach to the cross and forgiveness of the people who crucified him, is an excellent example to follow, learn from, and use to develop this moral virtue. This process of forgiveness takes place internally, without the need for permission from supervisors or workmates. It does not demand any religious involvement at work, especially since religious practices are realized outside of the workplace, being the private and intimate sphere of life. Workmates who share religious values can strengthen a forgiveness attitude and/or be role models in this issue.

As in Toussaint et al.’s (2018) research, the tendency to forgive was negatively linked to work stress. It has also been suggested that forgiveness can be considered from the perspective of an effective way of coping. This seems to be an appropriate direction to explain the relationship between religiosity and well-being at work. For example, results of Toussaint et al. (2018) study indicate that the relationship between forgiveness and some well-being indicators at work is partially mediated by stress. It is essential to determine how this moral virtue as a way of coping could be related to stress at work. Some suggestions are contained in ROSs (Pargament, 1997) as a significant moral instance that implies the intention and motivation to forgive as an attitude consistent with the declared worldview and values system.

The lack of forgiveness intention of an employee with a strong ROS can lead to cognitive dissonance (Festinger, 1957) and potential perception of themselves as an individual unable to forgive and acting inconsistently with religious demands. The desire to forgive positively impacts the perception of harm and wrongdoer intention by reevaluating it from the perspective of challenge, not treatment or harm, leading to not assigning the wrongdoer with bad intention and trying to look at the situation from the wider religious meaning perspective. It gives comfort in understanding the wrongdoer’s actions and facilitates the appearance of positive emotions, such as hope or compassion, instead of anger, resentment, and hate. It protects against hostility and the potential further anger rumination and reactive aggression (Quan et al., 2022) and closes the way to adjust positively. Another advantage of this kind of solution is the chance for reconciliation and building bonds between employees involved in this situation. On the other hand, in the case of an unforgiving attitude, negative moral emotions, such as hostility, generate tension and stress and force him/her to avoid transgressors, leading to a greater opportunity to erode a relationship. For example, previous research has indicated that hostility, as an attitude contrary to forgiveness, is harmful to physical health (Miller et al., 1996).

This study has some theoretical and practical implications. The mechanism of the relationship between religious practices and stress at work and disposition to forgive as a moral virtue underlying this link was confirmed. Prayer and mass attendance were correlated with a tendency to forgive, which in turn was linked with a lower level of stress at work. This study was the first attempt to verify the role of forgiveness as a moral virtue at work, which totally mediated the relationship between religious practices and stress at work. It has partially filled the gap regarding the role of religiosity in the workplace as an unnoticed and neglected topic in the literature that demands further exploration. The obtained results positively fit the image of the phenomenon of religiosity in the workplace to date, confirming its beneficial role among religiously committed people (Bickerton & Miner, 2021; Cunningham, 2014; Kutcher et al., 2010; Obregon et al., 2021; Qureshi & Shahjehan, 2021; Walker, 2013; Wnuk, 2022).

The achieved results from a practical point of view have paid attention to the role of religious involvement in the workplace, measured by religious practices, showing that this form of activity can be effective for dealing with stress because of a forgiving attitude.

It suggests for company owners, leaders, managers, and advisors (counselors) to respect employees’ religious beliefs and practices regardless of their denomination and shape the tolerance attitude toward individuals presented with other religious affiliations to counteract prejudice, labels, and victimization. Considering that this study was conducted in Poland, a very religious country with a homogenous denomination, where the rate of religiously active citizens is high and religious practices are common, it is recommended to encourage employees to develop their worldview based on religion as a factor positively related to forgiveness and, via this variable, lower levels of stress at work.

Tolerance for religiously skeptical and non-believers should be respected, trying to better recognize their needs and values at work, and within that strengthen the tendency to forgive and better cope with stress as a negative phenomenon at work.

Also, some training and workshops dedicated to learning how to forgive and showing that shaping this moral virtue can lead to positive work outcomes are needed. Recent studies have confirmed the beneficial effect of an intervention based on forgiveness on mental health and well-being in other populations than employees (López et al., 2021; Tao et al., 2020). Also, Worthington, et al. (2017) have emphasized the profits both at individual and societal levels as a consequence of promoting, shaping, and implementing a forgiveness attitude.

To fill this role, human resource (HR) representatives can be designated as individuals dedicated to taking care of the wide development of employees. Forgiveness is an interpersonal skill that should be developed, and this idea should be seen as important among HR managers and specialists focusing on employees’ development and well-being.

## Limitations and future research

The research conducted had some limitations, especially regarding generalizability and cross-sectional design. The results can be generalized only to the population of believer employees from Poland with the Roman Catholic Church affiliation. The next study should focus on representatives other than Roman Catholic denominations and encompass different cultural backgrounds, in particular, less religious populations, where religious socialization is not as common as in Poland, where religious values are not an important part of life, or where citizens have a hostile attitude toward religion. This could have shown whether the results of this study are independent of religious and cultural contexts. Previous research has indicated that the social–religious context is a factor determining the link between religiosity and well-being on the individual level. In this way, religious nations and religious people are satisfied and non-religious are not and inversely in more secular societies, non-religious representatives are happy contrary to religious individuals.

Considering that organizational culture reflects the national culture to a certain extent, it is possible that this pattern of religiosity–well-being connection is present among employees, especially since they are a significant part of the population. Only further multicultural studies could verify this point of view.

Future research on this topic should have a longitudinal character to define causality in the relationship between the study variables. Applied statistical methods present the direction of the relationship between the research variables. Still, other directions between the study variables should not be excluded due to the absence of tested alternative models. For example, even if previous studies have shown that religious involvement is an antecedent of forgiveness motivation, which in turn can serve as an effective way of coping with stress, it is possible that religiosity can be a consequence of a forgiving attitude, which leads to a lower level of stress.
